# Risk factors for venous thromboembolism in patients with lymphoma requiring hospitalization

**DOI:** 10.1038/s41408-018-0096-1

**Published:** 2018-06-07

**Authors:** Stefan Hohaus, Maria Chiara Tisi, Francesca Bartolomei, Annarosa Cuccaro, Elena Maiolo, Eleonora Alma, Francesco D’Alò, Silvia Bellesi, Elena Rossi, Valerio De Stefano

**Affiliations:** 1 Institute of Hematology, IRCCS Policlinico Gemelli Foundation, Catholic University of the Sacred Heart, Rome, Italy; 2 Division of Hematology, San Bortolo Hospital, Vincenza, Italy

Lymphoma is among the malignancies at high risk of venous thromboembolism (VTE)^1^. The VTE risk is the highest upfront during the first month after lymphoma diagnosis and decreases over time^2^. This upfront risk may be related to tumor burden and start of chemotherapy as contributing factors.

Routine assessment of thrombosis risk is recommended for patients with newly diagnosed neoplastic diseases^3^. Khorana et al developed a risk model for predicting chemotherapy-associated VTE based on baseline clinical and laboratory variables; however, only a minority of patients (12.6%) in the study cohort had lymphomas^1^. Several studies indicate a higher VTE risk in patients with aggressive non-Hodgkin lymphomas (NHL)^2,4–8^, advanced stage disease (III/IV)^2^, localization in the central nervous system (CNS)^9^, and use of anthracyclines^2^. The incidence of VTE during chemotherapy in NHL patients was investigated analyzing the databases of 12 Italian clinical trials, identifying DLBCL histology and Khorana score as risk factors^8^. A recent monocentric study identified mediastinal involvement, BMI > 30 kg/m^2^, reduced mobility, extranodal localization, development of neutropenia and hemoglobin level < 100 g/L as VTE risk factors in patients who had received at least one chemotherapy cycle^10^.

However, the inclusion criteria adopted in some of the aforementioned studies can produce an underestimation of the risk. Deriving risk scores from clinical trials and/or out-patient populations could produce results not tailored for lymphoma patients more prone to VTE because of a poorer clinical condition as suggested by the need for hospitalization. Hospitalization, in turn, is a risk factor for VTE in general and in cancer patients in particular^11,12^.

The aim of our study was to determine the incidence of VTE and to identify lymphoma-specific risk factors. We chose to investigate those patients with at least one hospital stay during the period of initial staging or subsequent therapy, in order to address this issue in a population with a reduced risk dilution.

Our study is a monocentric retrospective analysis of 857 adult patients with newly diagnosed lymphomas consecutively admitted and treated in our center from 2004 to 2015, and having had at least one hospital stay. The study was approved by our institutional review board. All patients provided written consent.

Diagnoses included the following: Diffuse large B cell lymphoma (DLBCL), *n* = 438; Hodgkin lymphoma (HL), *n* = 192; Follicular lymphoma (FL), *n* = 80; Peripheral T-cell lymphoma (PTCL), *n* = 61; Mantle cell lymphoma (MCL), *n* = 53; Primary CNS lymphoma (PCNSL), *n* = 33. DLBCL, PTCL, and MCL were labeled as aggressive lymphomas. Median age was 57 years (range 18–90). Other patient characteristics are shown in Table 1.

**Table 1:** Patient characteristics and VTE risk

		All VTE						Symptomatic VTE					
Parameter	Number of patients	Univariate analysis			Multivariate analysis			Univariate Analysis			Multivariate Analysis		
		OR	95% CI	*P* value	OR	95% CI	*P* value	OR	95% CI	*P* value	OR	95% CI	*P* value
Age > 60 years (*n* = 857)	384 (44.8%)	1.61	1.05–2.49	**0.03**	1.12	0.70–1.90	0.7	1.71	0.99–2.95	0.06			
Male gender (*n* = 857)	431 (50.3%)	1.05	0.69–1.61	0.8				1.19	0.70–2.04	0.5			
Aggressive histology^a^ (*n* = 857)	585 (68.3%)	2.17	1.23–3.72	**0.007**	0.74	0.53–1.03	0.08	2.61	1.26–5.40	**0.01**	1.10	0.47–2.60	0.8
PCNSL (*n* = 857)	33 (3.9%)	3.22	1.45–7.15	**0.004**	3.70	1.41–9.69	**0.008**		5.06	2.17–11.2	**<** **0.001**	6.32	2.21–18.2
Stage, III–IV (*n* = 849)	537 (63.2%)	1.34	0.84–2.13	0.2				0.89	0.51–1.55	0.7			
Bulk > 10 cm (*n* = 849)	208 (24.5%)	2.67	1.71–4.15	**0.001**	3.23	1.85–5.63	**0.0001**	2.04	1.17–3.58	**0.01**	2.84	1.42–5.72	**0.003**
ECOG ≥ 2 (*n* = 849)	270 (31.8 %)	3.28	2.10–5.10	**0.001**	1.80	1.03–3.13	**0.04**	4.26	2.39–7.60	**<** **0.001**	2.56	1.28–5.11	**0.008**
WBC > 11 × 10^9^/l (*n* = 831)	179 (21.5%)	1.31	0.80–2.15	0.3				1.70	0.93–3.08	0.08			
Plt > 350 × 10^9^/l (*n* = 832)	233 (28%)	0.83	0.50–1.36	0.5				0.48	0.23–1.0	0.05			
Hb < 10 g/dl (*n* = 839)	147 (17.5%)	1.06	0.61–1.85	0.8				1.34	0.70–2.61	0.4			
Albumin < 4 g/dl (*n* = 736)	417 (56.7%)	2.5	1.45–4.30	**0.001**	1.74	0.95–3.19	0.07	2.27	1.16–4.46	**0.02**	1.31	0.62–2.79	0.4
LDH elevated > UNV (*n* = 835)	314 (37.6%)	2.30	1.49–3.58	**0.001**	1.07	0.62–1.86	0.8	2.03	1.42–3.51	**0.01**	1.34	0.66–2.71	0.5

*P* values < 0.05 are shown in bold

Multivariate logistic regression analysis included 717 patients

*OR* odds ratio, *PCNSL* primary central nervous system lymphoma, *WBC* white blood cell count, *Plt* platelet count, *Hb* hemoglobin, *UNV* upper normal value

^a^Aggressive histology includes diffuse large B cell lymphoma, mantle cell lymphoma and peripheral T-cell lymphoma; follicular lymphoma and Hodgkin lymphoma were considered non-aggressive histology

We recorded all first objectively diagnosed deep vein thromboses (DVT) and/or pulmonary embolisms (PE). Thrombosis of superficial veins (*n* = 2) and of arteries (*n* = 8) was not considered an event of interest. Diagnosis of VTE was accepted only if it was confirmed by objective methods. VTE was classified as symptomatic when DVT and/or PE were associated with clinical signs or symptoms, and as incidental when routine imaging for disease evaluation revealed clinically asymptomatic events.

VTE was registered as heralding when was present at diagnosis before the start of treatment, and as treatment-related, when occurred during the first-line therapy in a time interval up to 9 months from the first cycle (i.e., in a time frame in which the first-line regimens consisting of 6–8 cycles of chemotherapy are usually completed).

Patients were followed from the time of diagnosis until the development of VTE, death, or loss to follow-up, whichever came first. The cumulative incidence of VTE was calculated from diagnosis according to the Kaplan–Meier method. VTE present at diagnosis were recorded as time 0. Risk factors for VTE were analyzed by univariate and multivariate analysis; a ROC analysis identified optimal cutoff points for continuous variables. In the competing risk analysis, we censored patients at the time of death. Competing-risk regression was based on Fine and Gray’s proportional subdistribution hazards model^13^. All tests were two-sided and *P* values < 0.05 were considered as significant. Statistical analyses were performed using the STATA 12 software (STATACORP, College Station, TX, USA).

Seventy-five patients did not complete the 9 months period and were censored at the time of last observation. During the entire observation period of 12,093 months (median 14 months per patient, range 6-15 months), 95 VTE events were observed. This corresponds to an overall rate of 11.1% (95/857); 18 patients had PE, that was isolated in 10 cases, and 11 had splanchnic venous thrombosis. In 54 patients, VTE was present at diagnosis or occurred until the first cycle of therapy, while in 41 VTE occurred during the first-line therapy. VTE was symptomatic in 57/95 patients (60%); heralding and treatment-related VTE were symptomatic in 35/54 (64.8%) and 22/41 (53.6%) patients, respectively (*p* = 0.29).

VTE rate differed with histology. PCNSL had a VTE peak incidence in PCNSL (9/33, 27.2%); further, the VTE rate was significantly higher in aggressive lymphomas (DLBCL = 12.6%, 55/438; PTCL 13.1%, 8/61; MCL 11.3%, 6/53; overall 13.3%, 78/585) than in HL (6.8%, 13/192), or FL (5%, 4/80) (overall 6.2%, 17/272) (*p* = 0.01). In the univariate analysis, age > 60 years, ECOG ≥ 2, aggressive histology, PCNSL, bulky disease > 10 cm, albumin levels ≤ 4.0 g/dL, and elevated LDH levels resulted significantly associated with VTE (Table 1).

The multivariate logistic regression analysis included only the factors with significance at the univariate analysis; ECOG ≥ 2, bulky disease > 10 cm, and PCNSL retained their significance as VTE risk factors (Table 1).

All characteristics that we identified as VTE risk factors with the exception of age were confirmed as risk factors for symptomatic VTE (Table 1). Most importantly, the multivariate analysis identified the same three risk factors (ECOG ≥ 2, bulky disease > 10 cm, and PCNSL) as risk factors for symptomatic VTE (Table 1).

Only a minority of patients (16%), in particular, those > 60 years and with a reduced performance status did not receive anthracyclines. Even after adjustment for such covariates, no association between use of doxorubicin and VTE was observed (data not shown).

We next grouped patients to the presence of risk factors. PCNSL patients had the highest VTE rate (27.2%). Patients with reduced performance status (ECOG ≥ 2) or bulky disease (>10 cm diameter) had a very similar VTE risk (53/270, 19.6% and 41/208, 19.7%, respectively), and were therefore grouped together in a group termed “bulk and/or ECOG” (69/377, 18.3%). The VTE rate in patients without these risk factors (*n* = 447) was 3.8%. The rate of symptomatic VTE was similar in the three risk groups (24.2% in PCNSL, 11.1% in the bulk and/or ECOG group, and 1.6% in the low-risk group). Figure 1 shows the risk over time in a competing-risk model. The curves suggest that the VTE risk factors are valid both for VTE events at diagnosis and during therapy.

**Fig. 1:**
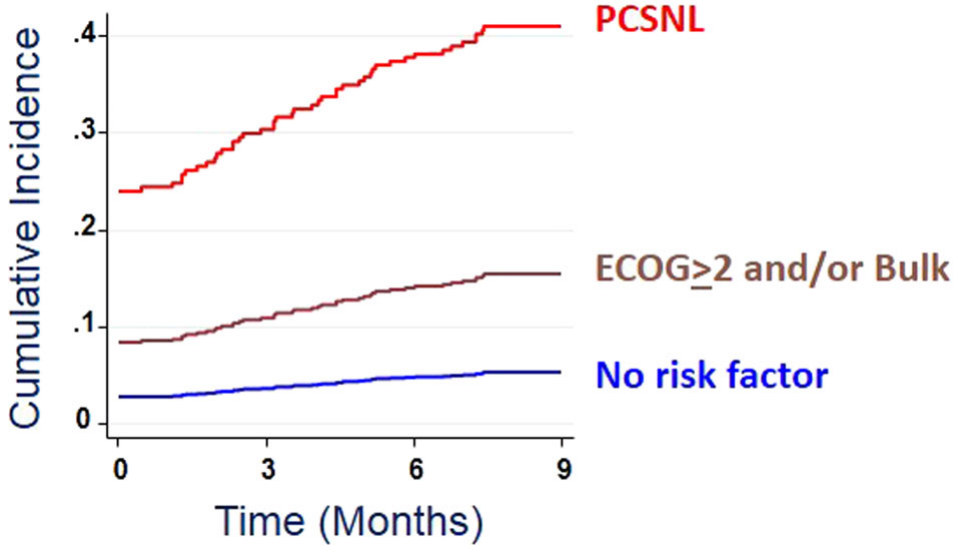
Cumulative incidence rates of VTE. The graph shows the results of a competing risk regression analysis. Death was counted as a competitive event. VTE before the start of systemic therapy were considered heralding events and were counted as events at time 0, while VTE during the first nine months from treatment start was considered treatment-related events. Subdistribution hazard risk was 5.2-fold (95% CI, 3.1–8.8) for patients with the risk factors reduced performance status (ECOG > 2) and/or bulky disease (>10 cm), and 8.0-fold (95% C.I., 3.7–17.5) for patients with PCNSL.Analysis of the treatment-related VTE in 803 patients, after exclusion of the 54 ones who presented with VTE before the start of therapy, confirmed a VTE risk gradient similar to that of the overall cohort (see text) (5/29, 17.2% in PCNSL, 25/333, 7.5% in the bulk and/or ECOG group, and 11/441, 2.5% in the low-risk group)

An analysis focusing on patients with score 0 did not identify a risk factor possibly discerning patients at lowest risk (data not shown).

In conclusion, we identified VTE risk factors in patients with lymphoma that are well different from VTE risk factors described for solid tumors. We identified three main clinical risk factors: CNS localization, tumor bulk > 10 cm and reduced performance status. We confirm aggressive lymphoma histology as VTE risk factor in univariate, but not in multivariate analysis.

Bulk and reduced ECOG probably contribute to slowing down the blood flow in the venous bed. In fact, analyzing for associations between localization of VTE and risk factors, we found an association between the site of the bulk and thrombosis (data not shown). A reduction of the performance status to the ECOG grade 2 or greater that defines a reduction of the daily activity with variation from partial to complete immobilization was associated with a higher VTE localization to the lower extremities.

Analyzing for laboratory abnormalities, we found lower albumin and elevated LDH levels at diagnosis to be associated with VTE. LDH is a proliferation marker typically elevated in aggressive lymphomas. Low albumin levels may be due to an altered nutritional status or an inflammatory environment reducing albumin production and may lead to endothelial cell activation increasing the thrombotic risk^14^. Other laboratory abnormalities, in particular, the CBC counts that are an essential part of the Khorana score, did not prove to be significant in lymphoma patients.

Comparison with other studies^8,10^ is difficult because of differences in patient selection. Our study cohort is a real-life case series including both HL and NHL, including also VTE that was present before the start of therapy. This is well justified as the recent analysis of a Swedish registry of 40,354 NHL patients showed that the incidence of thrombosis started to increase about five months before diagnosis, and reached its peak a month before diagnosis^15^. We included only patients with at least one hospital stay. Patients as those with low-grade histology who did not require a hospital stay were not retrieved. As expected, this may explain the higher incidence rate of VTE with respect to other reports^1,2,8,10^. Therefore, our study tackles the issue of VTE frequency in a population at increased risk, in whom special care is required and specific information are needed.

In summary, we identified lymphoma-specific VTE risk factors that could be useful tools to plan tailored antithrombotic prophylaxis.

## References

[1] Khorana AA, Kuderer NM, Culakova E, Lyman GH, Francis CW (2008). Development and validation of a predictive model for chemotherapy-associated thrombosis. Blood.

[2] Sanfilippo KM (2016). Incidence of venous thromboembolism in patients with non-Hodgkin lymphoma. Thromb. Res..

[3] Lyman GH (2013). Venous thromboembolism prophylaxis and treatment in patients with cancer: American Society of Clinical Oncology clinical practice guideline update. J. Clin. Oncol..

[4] Caruso V (2010). Thrombotic complications in adult patients with lymphoma: a meta-analysis of 29 independent cohorts including 18 018 patients and 1149 events. Blood.

[5] Mohren M (2005). Increased risk of thromboembolism in patients with malignant lymphoma: a single-centre analysis. Br. J. Cancer.

[6] Park LC (2012). Incidence, risk factors and clinical features of venous thromboembolism in newly diagnosed lymphoma patients: results from a prospective cohort study with Asian population. Thromb. Res..

[7] Lund JL, Østgård LS, Prandoni P, Sørensen HT, de Nully Brown P (2015). Incidence, determinants and the transient impact of cancer treatments on venous thromboembolism risk among lymphoma patients in Denmark. Thromb. Res..

[8] Santi RM (2017). Khorana score and histotype predicts incidence of early venous thromboembolism in non-Hodgkin lymphomas. A pooled-data analysis of 12 clinical trials of Fondazione Italiana Linfomi (FIL). Thromb. Haemost..

[9] Goldschmidt N, Linetsky E, Shalom E, Varon D, Siegal T (2003). High incidence of thromboembolism in patients with central nervous system lymphoma. Cancer.

[10] Antic D (2016). Development and validation of multivariable predictive model for thromboembolic events in lymphoma patients. Am. J. Hematol..

[11] Heit JA (2002). Relative impact of risk factors for deep vein thrombosis and pulmonary embolism: a population-based study. Arch. Intern. Med..

[12] Prandoni P, Samama MM (2008). Risk stratification and venous thromboprophylaxis in hospitalized medical and cancer patients. Br. J. Haematol..

[13] Fine JP, Gray RJ (1999). A proportional hazards model for the subdistribution of a competing risk. J. Am. Stat. Assoc..

[14] Falanga A, Marchetti M, Russo L (2015). The mechanisms of cancer-associated thrombosis. Thromb. Res..

[15] Birgisdóttir AM, Sverrisdóttir IS, Landgren O, Björkholm M, Kristinsson SY (2017). Risk of thrombosis in patients with non-Hodgkin’s lymphoma: a population-based cohort study. Haematologica.

